# Does parathyroid autofluorescence reduces unintentional parathyroidectomy during total thyroidectomy with central lymph node compartment dissection?

**DOI:** 10.1007/s13304-026-02762-z

**Published:** 2026-07-15

**Authors:** Angeliki Chorti, Ioannis Pliakos, Moysis Moysidis, Sohail Bakkar, Theodossis Papavramidis

**Affiliations:** 1 Department of Surgery, AHEPA University Hospital, Aristotle University of Thessaloniki, Gr Stefanou 14, 54352 Thessaloniki, Greece; 2 Unit of Minimally Invasive Endocrine Surgery, Kyanous Stavros, Thessaloniki, Greece; 3 Department of Surgery, Faculty of Medicine, the Hashemite University, Zarqa, 13131 Jordan

**Keywords:** Autofluorescence, Total thyroidectomy, Thyroid cancer, Central lymph node dissection, FLUOBEAM^®^ LX

## Abstract

Postoperative hypoparathyroidism is the most frequent complication after thyroid surgery. Parathyroid autofluorescence ability serves as a useful tool for localizing parathyroid glands intraoperatively. The aim of this study is to evaluate the efficacy of intra-operative autofluorescence imaging concerning the unintentional excision rate of parathyroids during total thyroidectomy with central lymph node compartment dissection. This s a prospective randomized study enrolling patients with thyroid cancer that underwent total thyroidectomy with therapeutic bilateral central lymph node compartment dissection in a high-volume center during a two-year period. Patients were separated in two groups [without (A) or with FLUOBEAM^®^ LX (B)] and the number of identified parathyroid glands intraoperatively was recorded and correlated with postoperative levels of parathyroid hormone and serum calcium. One-hundred eighty patients were included in this study. A higher number of parathyroid glands were identified and distinguished from lymph nodes in the central compartment with autofluorescence compared to naked eye visualization (3.84 ± 0.51 vs. 3.69 ± 0.78, *p* = 0.03). The number of parathyroid glands found in specimen were lower in Group B (17 vs. 8, *p* = 0.06). Group B had lower incidence of transient postoperative hypoparathyroidism (16.1% vs. 9.4%, *p* = 0.058, odds ratio 0.54, 95% CI 0.28–1.02). Moreover, the autofluorescence score had influence on postoperative transient hypoparathyroidism rates (*p* < 0.001). Parathyroid autofluorescence is a safe and efficient tool in experienced hands during central compartment neck dissection, improving postoperative hypoparathyroidism rates and quality of lymph node dissection.

## Introduction

Postoperative hypoparathyroidism (PH) remains a significant problem after thyroid surgery. The incidence of PH varies in the international literature, but, according to a meta-analysis, temporary hypoparathyroidism accounts for 19–38% and permanent hypoparathyroidism for 0–3% [1, 2].

The mechanisms that underlie surgery-related hypoparathyroidism are related to disruption of parathyroid arterial supply or venous drainage, mechanical injury, thermal or electrical injury, and either intentional or inadvertent partial or complete removal [1, 3, 4].

Autofluorescence is the ability of several natural substances to emit light. It has been already proved that parathyroid glands emit their own light after near-infrared (NIR) around 820 nm, providing high contrast to the surrounding tissues. This made near-infrared autofluorescence a potential useful tool in hands of experienced endocrine surgeons in order to distinguish parathyroid glands from other anatomic structures during thyroidectomies [5].

The aim of the present study is to evaluate the efficacy of intra-operative autofluorescence imaging concerning the unintentional excision rate of parathyroids during total thyroidectomy with central lymph node compartment dissection. Moreover, the correlation of autofluorescence with 24 h post-operative parathyroid hormone (PTH) and serum calcium levels will be evaluated.

## Methods

### Study design

This study was designed as a randomized prospective study. Patients with thyroid cancer that underwent total thyroidectomy with therapeutic bilateral central lymph node compartment dissection (CCLND) in a high-volume center during a 2-year period were enrolled in this study. Inclusion criteria were: (a) age over 18-years old, (b) patient scheduled for a non-emergency operation and (c) patient eligible for total thyroidectomy. Exclusion criteria were: (a) patient is participating in another clinical trial which may affect this study’s outcomes, (b) prior operation in the neck, (c) primary or secondary hyperparathyroidism, (d) vitamin D deficiency and (e) use of drugs that influences calcium metabolism (Vitamin D analogues, oral calcium supplements, bisphosphonates, teriparatide, thiazide diuretics, aromatase inhibitors). Participants were allocated randomly in 1:1 ratio to two groups: Group A (total thyroidectomy with CCLND without application of autofluorescence device) and Group B (total thyroidectomy with CCLND with application of autofluorescence device). In this study, autofluorescence camera-based system, FLUOBEAM^®^ LX (Fluoptics, Grenoble, France) was applied as parathyroid near-infrared autofluorescence detector device. The primary endpoint of this study is to evaluate the use of autofluorescence to distinguish parathyroid glands during thyroidectomy with central lymph node dissection. The secondary endpoints are to correlate postoperative hypoparathyroidism levels with the application of autofluorescence device and to detect the changes of practice in performing total thyroidectomy with central lymph node dissection when monitoring parathyroids with autofluorescence.

Minimally invasive total thyroidectomy with therapeutic central lymph node compartment dissection was performed by the same high-volume endocrine surgeon (> 300 procedures per year). A small neck incision (2 cm) was performed and blunt dissection of strap muscles followed. The superior thyroid vessels were ligated, the recurrent laryngeal nerve was detected and then, the inferior vessels were also ligated. The thyroid lobe was excised and isthmus and possible pyramidal lobe dissection followed. The same procedure was performed for the remaining thyroid lobe. Afterwards, bilateral central compartment lymph node dissection was performed. In group A, parathyroid gland identification was only based on naked eye visualization, while in group B parathyroid glands detection was confirmed by autofluorescence application.

### Data collection

Patients’ clinicopathological data (age, gender, pre- and postoperative biochemical data, pathology report including number of excised parathyroid glands) have been recorded. Furthermore, in Group B the number of visualized parathyroid glands using FLUOBEAM^®^ LX have been also recorded. The intraoperative autofluorescence score was defined as 0 when there was no gland visualization, 1 when one gland was detected and 2 when at least 2 parathyroid glands were visualized. Hypoparathyroidism was defined as PTH < 15 pg/ml and hypocalcemia as serum albumin-corrected calcium levels < 8.5 mg/dl in 1st postoperative day.

Data collection and analysis were performed according to the institute’s scientific policy that ensures maintaining data confidentiality and the ethical standards of the Helsinki declaration 1975 and its later amendments and comparable ethical standards. A written approval was obtained (32586/6.4.2020) by the Local Ethical Committee. Patients’ informed consent was also obtained by each participant.

### Statistics

Quantitative variables that follow a normal distribution were reported as means ± standard deviation or median (interquartile range) for those that don’t follow a normal distribution. Qualitative variables were reported as frequencies. Sample normality was tested using the Shapiro-Wilk test. If the continuous variables didn’t follow a normal distribution, non-parametrical tests were used. To compare the median values of the quantitative variables in the categories of binary categorical variables, the Mann-Whitney test was used and for mean values in normal distribution, independent sample t-test was applied. Finally, with the X^2^ test and Fisher’s exact test (between categorical variables), possible correlations and dependencies between the variables were investigated. The statistical processing of the sample was done using the statistical package “Statistical Package for the Social Sciences (SPSS)” version 26 (IBM), (SPSS Inc., Chicago, IL, USA). In all cases, a *p*-value < 0.05 was set as the level of statistical significance for a two-tailed test.

## Results

In this study, 180 patients that underwent total thyroidectomy with therapeutic bilateral central lymph node compartment dissection were assigned to each group (Group A and B). No losses or exclusions occurred during follow-up. The clinicopathological features of each group are demonstrated in Table 1. There was no statistically significant difference among groups in terms of demographic and clinical characteristics.

**Table 1:** Clinicopathological characteristics of patients enrolled (PTH: parathyroid hormone)

	Group A (without FLUOBEAM)	Group B (with FLUOBEAM)	*P*-value
Age (years old)	49.8 ± 13.9	49.3 ± 13.7	0.62
Gender (male: female)	33:147	44:136	0.15
Preoperative PTH (pg/ml)	50.25 ± 16.82	48.34 ± 15.12	0.19
Preoperative serum calcium (mg/dl)	9.52 ± 0.51	9.5 ± 0.5	0.67
Postoperative PTH (pg/ml)	22.9 ± 12.2	25.86 ± 14.48	**0.06**
Postoperative serum calcium (mg/dl)	8.76 ± 0.48	8.73 ± 0.53	0.93

A higher number of parathyroid glands were identified with autofluorescence compared to naked eye visualization (3.84 ± 0.51 vs. 3.69 ± 0.78, *p* = 0.03) (Table 2). Especially in the central compartment lymph node dissection, the distinction between metastatic lymph nodes and parathyroid glands improved with the application of FLUOBEAM^®^ LX. The number of parathyroid glands found in specimen were lower in Group B (17 vs. 8, *p* = 0.06), but there was not statistically significant difference.

**Table 2:** Parathyroid glands identified in both groups

	Group A (without FLUOBEAM)	Group B (with FLUOBEAM)	*P*-value
No of identified parathyroid glands	3.69 ± 0.78	3.84 ± 0.51	0.03
Parathyroid specimen in pathology report (< 2 glands)	17	8	0.06

**Fig. 1:**
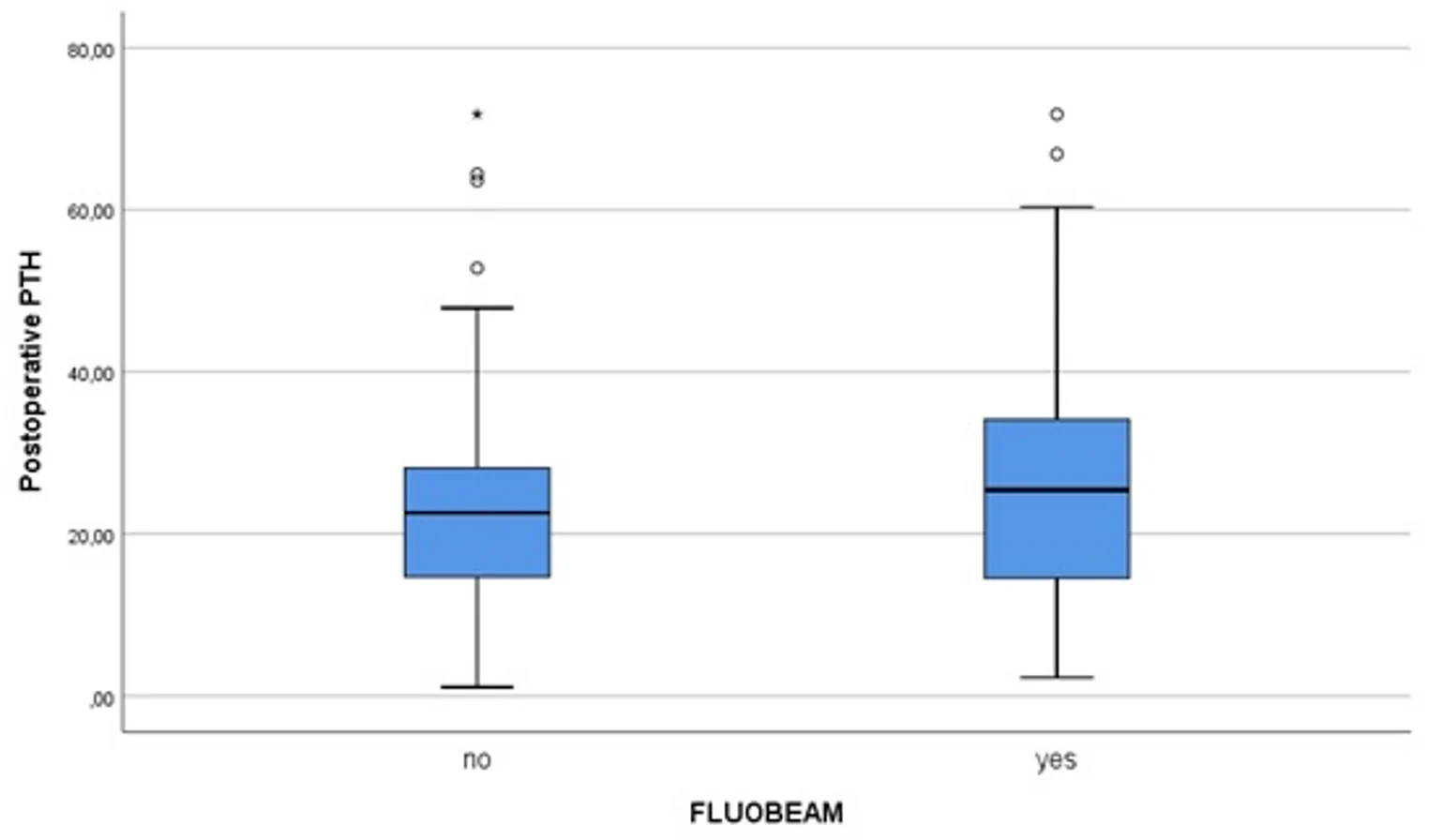
Difference of postoperative parathyroid hormone levels among groups (*p* = 0.06)

We identified a tendency of statistically significant difference of transient postoperative hypoparathyroidism among groups, favoring Group B with lower incidence (16.1% vs. 9.4%, *p* = 0.058, odds ratio 0.54, 95% CI 0.28–1.02). The rate of postoperative transient hypocalcemia did not differ among groups (*p* = 0.93), but we found a tendency for postoperative levels of serum PTH to be lower in Group B (22.9 ± 12.2 vs. 25.86 ± 14.48, *p* = 0.06) (Fig. 1).

**Fig. 2:**
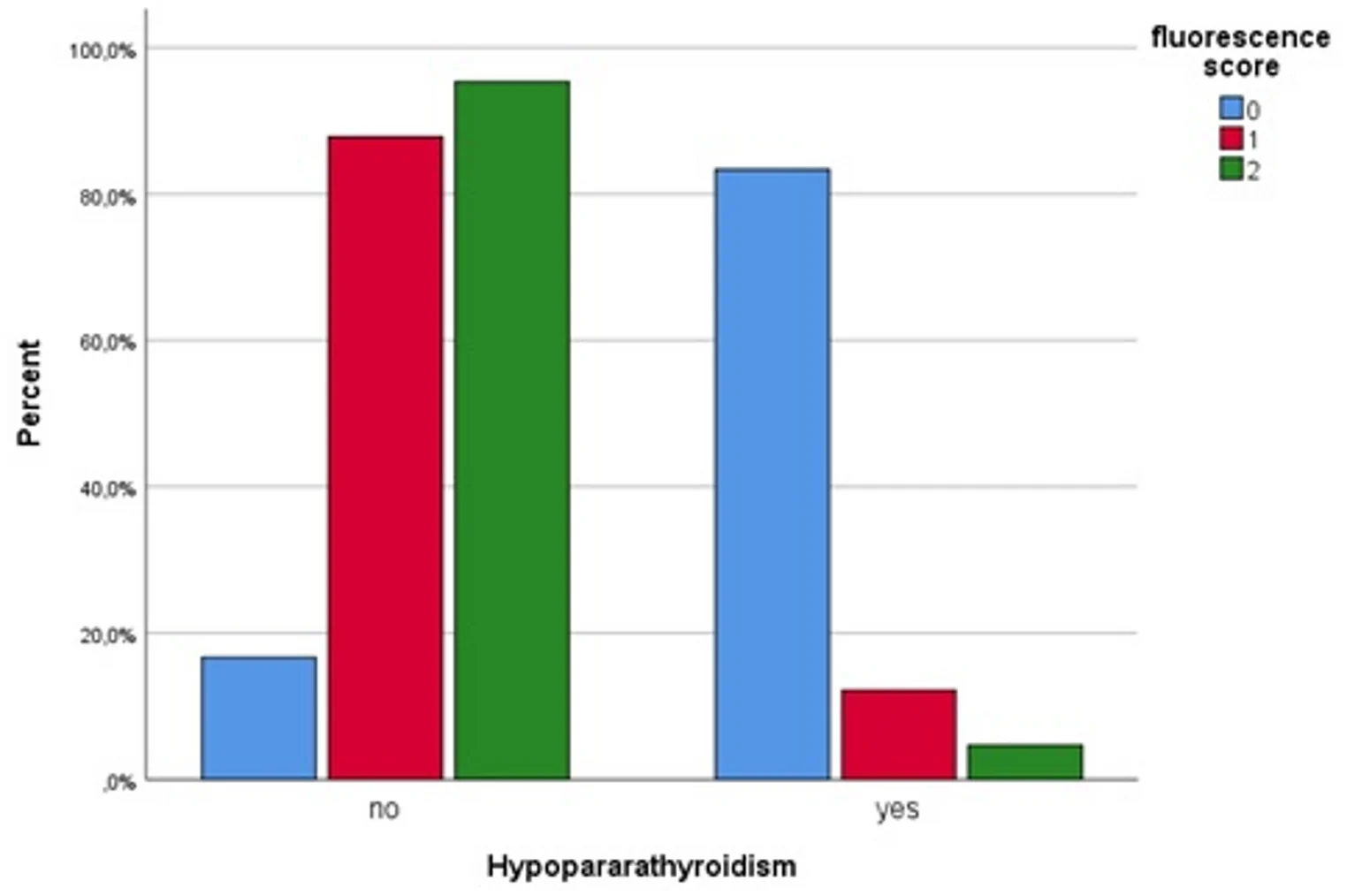
Correlation of autofluorescence score with postoperative hypoparathyroidism (*p* < 0.001)

The rates of intraoperative fluorescence score for Group B were: 6.7% for score 0, 45.6% for score 1 and 47.8% for score 2. The correlation between fluorescence score and the presence of parathyroid glands in specimen was statistically significant (*p* = 0.007). Moreover, the autofluorescence score had influence on postoperative transient hypoparathyroidism rates (*p* < 0.001). As the autofluorescence score increased, the rate of postoperative hypoparathyroidism declined (Fig. 2). Moreover, no specific harm to the patient has been observed with the application of FLUOBEAM^®^ LX.

## Discussion

Postoperative hypoparathyroidism serves as the most frequent complication after thyroid surgery. Parathyroid gland auto transplantation has been applied to tackle this problem, but a recent study revealed no decrease in rate of permanent hypoparathyroidism [6]. The introduction of parathyroid gland autofluorescence ability led to the development of camera-based or probe-based devices that can detect parathyroid tissue intraoperatively [7]. A randomized controlled trial, comparing white surgical light and near-infrared autofluorescence during total thyroidectomy, resulted that the rate of identified parathyroid glands increased with the insertion of autofluorescence devices and thus at least 1 previously missed parathyroid gland was identified in 67% of cases [8]. An Australian study confirmed the superiority of near-infrared autofluorescence devices to detect parathyroid glands compared to direct visualization [9]. A previous study proved the benefit of near-infrared autofluorescence on parathyroid gland intraoperative recognition and preservation during total thyroidectomy, but it failed to predict postoperative transient hypoparathyroidism [10]. This was also supported by other investigators, that also proved the correlation of intraoperative autofluorescence application with reduction in postoperative hypoparathyroidism [11–13]. Four recent meta-analyses also confirmed the decreased risk for postoperative hypocalcemia with the application of camera-based autofluorescence [14–17]. Barbieri et al. stated the reduction of oral calcium supplementation postoperatively with the use of near-infrared autofluorescence [12]. The addition of indocyanine green to near-infrared autofluorescence seemed to improve parathyroid gland detection, prevent devascularization and mitigate postoperative hypoparathyroidism [18, 19]. Parathyroid autofluorescence was considered better in localizing parathyroid glands compared to indocyanine green angiography that excelled in assessing parathyroid perfusion [20]. In two consecutive meta-analyses, the sensitivity and specificity of near-infrared autofluorescence were found 97–98% and 93–99%, respectively [21, 22].

Near-infrared autofluorescence can be more useful in terms of central compartment lymph node dissection, as parathyroid glands can be detected and distinguished from the surrounding lymph nodes, resulting in better lymph node dissection quality and preservation of parathyroid glands that could be inadvertently excised. In our study, the application of a camera-based system, FLUOBEAM^®^ LX, found to be an efficient tool in experienced hands for the intraoperative identification of parathyroid glands. During total thyroidectomy with central compartment lymph node dissection, a significantly higher number of parathyroid glands were detected in autofluorescence group compared to the control group. Bakkar et al. also reported a higher rate of parathyroid detection with autofluorescence application (100% vs. 81%, *p* < 0.01) in cases of therapeutic bilateral central compartment lymph node dissection [23]. Furthermore, the number of excised lymph nodes may be higher with the use of FLUOBEAM^®^ LX, resulting in better central neck dissection quality [24]. Our study concluded that the number of parathyroid glands found in pathology specimen were lower in autofluorescence group, but a statistically significant difference was not found among groups. A statistically significant difference in the total number of excised parathyroid glands was found by Kim et al., with the autofluorescence group scoring less [25]. A lower number of unintentionally excised parathyroid glands with the use of FLUOBEAM^®^ LX is reported in the study by Bergenfelz et al. [26]. Romero-Velez et al. found that the rate of parathyroid glands detected in specimen was decreased when near-infrared autofluorescence was used for identification and confirmation of parathyroid glands and not only for confirmation [27].

Moreover, in our study, the rates of postoperative hypoparathyroidism in autofluorescence group showed a downward tendency in comparison to control group, although without statistically significant difference. The postoperative levels of serum PTH seemed to be affected after the introduction of autofluorescence, whereas postoperative levels of serum calcium were similar among groups. This trend has been also identified in other studies, which concluded that the overall incidence of postoperative transient hypoparathyroidism was not minimized after the insertion of autofluorescence [23, 28]. Lykke et al. stated that hypoparathyroidism was lower in autofluorescence group but not statistically significant [29]. Lower postoperative levels of serum PTH without hypocalcemia was also found by Kim et al. after total thyroidectomy with CCND [25]. Conversely, Bergenfelz et al. [26] found low levels of serum PTH in 1st postoperative day after total thyroidectomy and CCND in 33% of control group versus 18% of FLUOBEAM^®^ LX group (*p* = 0.021). Furthermore, the rate of transient postoperative hypocalcemia was correlated with the application of autofluorescence. Postoperative levels of serum albumin-corrected calcium were lower in autofluorescence group compared to the control group (36% vs. 60%, *p* = 0.003) [30].

Based on our study, camera-based near-infrared autofluorescence devices are useful tools for the intraoperative detection of parathyroid glands and, consequently, the reduction of postoperative transient hypoparathyroidism rates in high-volume centers. A study conducted in a high-volume center supported that the incidence of postoperative hypoparathyroidism in high-volume centers is low and further reduction by the application of technological adjuncts cannot be evaluated, as the elevated cost and complexity of these systems restrict the routine use [31]. Conversely, low-volume centers seem to benefit more from the introduction of parathyroid autofluorescence, as they resulted in a decrease of postoperative transient and permanent hypoparathyroidism rates [32]. Early-career endocrine surgeon may exploit more by the use of autofluorescence than mid-career surgeons [27]. In multicentric study, only 42% of surgeon supported that near-infrared autofluorescence detected parathyroid glands before they became apparent and 45% that it ameliorated parathyroid gland preservation during central neck compartment dissection [33].

This study harbored several limitations. First of all, the autofluorescence signals evaluation was performed by the surgeon’s objective opinion and cannot be quantified. Moreover, the sample size of this study was relatively small and further larger studies are mandated to evaluate thoroughly the efficacy of near-infrared autofluorescence during total thyroidectomy with central neck compartment dissection.

## Conclusion

Camera-based near-infrared autofluorescence devices are useful tools also in experienced hands for the identification and confirmation of parathyroid glands during total thyroidectomy with central compartment lymph node dissection. We confirmed the utility of this technology on the prevention of unintentional parathyroid gland excision. The reduction in postoperative transient hypoparathyroidism has not been proved statistically significant, although it showed a downward trend. Further larger studies are mandatory to reinforce these results. 
